# When is genetic modification socially acceptable? When used to advance human health through avenues other than food

**DOI:** 10.1371/journal.pone.0178227

**Published:** 2017-06-07

**Authors:** Nicole J. Olynk Widmar, S. R. Dominick, Wallace E. Tyner, Audrey Ruple

**Affiliations:** 1 Dept. of Agricultural Economics, Purdue University, West Lafayette, Indiana, United States of America; 2 Dept. of Comparative Pathobiology, Purdue University, West Lafayette, Indiana, United States of America; Texas A&M University College Station, UNITED STATES

## Abstract

Given the potential for genetic modification (GM) to impact human health, via food and health mechanisms, a greater understanding of the social acceptance of GM is necessary to facilitate improved health outcomes. This analysis sought to quantify U.S. residents’ acceptance of GM across five potential uses (grain production, fruit or vegetable production, livestock production, human medicine, and human health, i.e. disease vector control) and provides an in-depth analysis of a timely case study–the Zika virus (ZIKV). The two categories with the highest levels of acceptance for GM use were human medicine (62% acceptance) and human health (68% acceptance); the proportions agreeing with the use of GM for these two categories were statistically different from all other categories. Acceptance of GM in food uses revealed 44% of the sample accepted the use of GM in livestock production while grain production and fruit and vegetable production showed similar levels of agreement with 49% and 48% of responses, respectively. Two variables were significant in all five models predicting GM acceptance; namely, being male and GM awareness. Being male was significant and positive for all models; respondents who reported being male were more likely (than those who reported female) to agree with all five of the uses of GM studied. Those who were reportedly aware of GM mosquito technology were also more likely to agree with all uses of GM technology investigated. The potential relationship between awareness of GM technology uses and acceptance of other uses could help inform rates of acceptance of new technologies by various population segments.

## Introduction

Genetic modification (GM) of plants and animals by humans has occurred for centuries through the process of domestication and conventional breeding. However, GM of field crops, particularly insect resistant corn and herbicide tolerant soybeans, has recently been the source of controversy in the public sphere. Some public intellectuals outside of agriculture have taken sides in the debate on GM crops including the economist Nassim Taleb [1], biologist Richard Dawkins [2], and philosopher Peter Singer [3]. Some lines drawn were that the health risks are unpredictable [1], the real risk is in the combination and use [2] and that risks of one form of use (agriculture) should be evenly distributed to (or also ignored in) other uses (pharmaceuticals) [3]. Even then, scientific evidence suggests that GM crops are not dangerous, and the evidence from economics shows that GM crops are associated with positive economic outcomes, including for the poorest people [4]. Nonetheless, many consumers demonstrate a preference for non-GM crops.

Certainly the public perception or acceptance of technologies, including GM technologies, has the potential to shape public policy, impact investments in research and development, and ultimately influence the development and use of technologies in society. In general, public perceptions of the use of GM within the context of medicine have been largely favorable [5]. Technological advances in the 1970s allowed an expedited process of genetic engineering through direct manipulation of the genome, which rapidly led to development of synthetic hormones, such as somatostatin, and medications, like insulin [6][7]. Use of genetic engineering also had a dramatic effect on vaccine development, and recombinant vaccines were created for diseases like hepatitis B, Lyme disease, cytomegalovirus, and pertussis [8][9][10][11]. While advances in the development of medications and vaccines may be enabled by the use of genetic engineering the degree to which the public, or specific members of the public, recognize genetic engineering in these advances remains elusive in many cases. For example, even if someone is well-informed with regard to medical advances and aware of the need for advances in vaccine development, do they necessarily recognize the role that genetic engineering has in those advances?

The recent outbreak of Zika virus (ZIKV) in the Americas and Caribbean was declared a Public Health Emergency of International Concern by the World Health Organization [12]. The location of the 2016 Summer Olympics in a region that was highly impacted by ZIKV, Brazil fueled what was already significant coverage of the outbreak. Symptoms associated with the ZIKV epidemic in Brazil range from microcephaly in fetuses and newborns [13] and death in some patients [14], in addition to the constellation of other milder symptoms previously reported, such as rash, fever, and headache [15]. There currently exists the option of using a GM mosquito to mitigate the spread of ZIKV. The GM mosquito (Aedes Aegypti) which mates to bear terminal offspring could be employed to fight the spread of ZIKV by being released to mate, thereby baring terminal offspring and significantly reducing the mosquito population. It is not currently known if the U.S. public will consider the use of GM of mosquitoes ethical in order to mitigate the spread of ZIKV in various regions of the World (including within the U.S.). According to a 2016 report, Florida residents favor the use of GM mosquitos, 60% compared to 50% general favor by the remaining U.S. [16]. Understanding acceptance of the use of GM mosquitoes to combat the ZIKV outbreak may help provide insight into likely acceptance of GM technologies to combat other, potentially unforeseen, health crises.

This analysis sought to quantify U.S. resident’s acceptance of GM across five potential categories or uses, including grain production, fruit or vegetable production, livestock production, human medicine, and human health reasons (i.e. disease vector control such as the GM mosquito to control ZIKV). The five GM uses studied in this analysis were selected to allow comparisons between direct impacts on human health (through human medicine and human health factors i.e. disease vector control) and the more indirect impacts on human well-being through food production (including grain production, fruit or vegetable production, and livestock production). Given the press and media attention on some aspects of GM use (particularly outside of medicine), it is hypothesized that significantly larger proportions of survey respondents will accept GM in medical uses than the other uses studied. The timing of the outbreak relative to data collection for this study facilitates ZIKV as a case study or specific example for study in this analysis. Currently, health officials and leaders worldwide are struggling to address ZIKV and deal with the devastating human health impacts being realized. Given the ZIKV challenges being faced, this analysis delves specifically into the acceptance of GM mosquitoes to help combat ZIKV. The fundamental contribution of this analysis is the identification of significant factors in predicting acceptance of GM across the five uses, which included both food and non-food uses impacting human health.

## Materials and methods

### Survey development and administration

On February 10^th^, 2016 a web-based survey, hosted on Qualtrics, was launched in order to understand respondent’s acceptance of GM uses. The survey instrument, in its entirety, is provided as a supplementary file S2 File. Data collection concluded on the 12^th^ of February. Lightspeed GMI provided a panel of opt-in respondents, and 964 completed surveys were collected from U.S. residents (data provided in S1 File). Quotas were set within Qualtrics to facilitate the collection of a sample of respondents which was targeted to be representative of the U.S. population in terms of age, gender, income, education, and region of residence in accordance with the 2014 U.S. Census Bureau estimates [17].

Respondents were asked basic demographic questions, including questions about recent travel (focusing on the Caribbean) and general ZIKV awareness. Central to the objective of this analysis, inquiries were made into respondent’s perceptions and beliefs about acceptable uses for GM technology. Specifically, the question read *Please indicate whether you agree or disagree with the following uses of GM* and respondents were provided the following list: grain production, fruit or vegetable production, livestock production, human medicine, and human health reasons (i.e. disease vector control). Respondents were asked to select “strongly disagree”, “disagree”, “agree”, or “strongly agree” in response to each use provided.

Given the timing of the data collection and intention to incorporate respondent’s perceptions of ZIKV as an example of a potential GM-based solution to a human health challenge, questions regarding mosquito control mechanisms were asked. Questions asked gathered information on respondent’s knowledge and perceptions of mosquito borne illnesses, preferred mosquito control methods in the Caribbean and U.S., and awareness and acceptance of GM mosquitoes in the U.S. and Caribbean.

Basic summary statistics were calculated for all demographics collected and for all GM acceptance and understanding questions. The Fisher’s Exact test, which directly calculates a p-value, was used to compare the proportions of the sample reporting acceptance of GM uses and was conducted in STATA 14.0. The Fisher’s Exact test tests against the hypothesis that the proportions of individuals who accept different uses of GM technology are not statistically different. Cross tabulations were used to understand relationships between demographics, GM acceptance and knowledge levels about GM. Further, pairwise correlations were used to understand the relationship between agreement in one category of GM use and agreement in another.

### Ordered logit models

Five maximum likelihood ordered logit models were estimated with respect to the respondent’s agreement with the use of GM across the five categories (uses) of grain production, fruit or vegetable production, livestock production, human medicine, and human health reasons (i.e. disease vector control). The five GM uses studied were provided in the survey without definition and interpretation was left up to the respondent. This analysis focused on the public perceptions of GM uses, and asked specifically about acceptance of specific mosquito control mechanisms. Often residents are asked to weigh in on issues, such as the GM mosquito, with limited background or supplementary information readily available. Furthermore, residents often form perceptions or opinions with limited, or varying, interpretation of various uses or technologies. Thus, respondents were presented with each of the five GM uses, but not provided with background information or details surrounding those uses.

The nature of the debate surrounding GM uses and the rather discrete paths forward surrounding GM uses, of either allow or not allow usage, make a discrete response surrounding GM use most easily/directly interpretable. In order to facilitate interpretation of responses with regard to acceptance of GM uses, the responses were used as discrete ranks for ordered logit estimation. For each of the five uses a discrete dependent variable was created with (1) being “strongly disagree”, (2) “disagree”, (3) “agree”, and (4) “strongly agree” The probability of respondent(s) increasingly agreeing with the use of GM technology for a particular category can be estimated and is represented using ordered logits.

Ordered logits are an appropriate analysis tool for this dependent variable. The nature of the variable is categorical or discrete and the numerical ranks are superficial and only establish that (1) or “strongly disagree” is below (2) “disagree” which is below (3) “agree”, and (4) “strongly agree”, when estimating how likely respondents are to agreeing with the presented uses of GMO technology. That is to say, agreement of 1.5 or 3.76 is meaningless because no discrete category was assigned to those values. Wooldridge [18] explains that dependent variables can be limited to a small number of values and are discrete (the fractions are meaningless or inappropriate) and, there for, continuous estimators can have limitations. The ranked nature of the dependent variable also makes linear or cardinal regressions inappropriate but discrete ordered dependent variables, such as Likert scale values, can be regressed using ordered logits [19]. Other studies have used ordered logits to analyze Likert scale developed dependent variables [20][21].

Using the ranked categorical dependent variables thresholds are generated. The ordered logit estimates the likelihood an outcome would fall between or beyond the threshold [19]. With *y* representing the dependent variable; *y* = 1, 2, 3, 4, and using *k* to represent thresholds, *j*, a rank would be established in the following way

$$If\;k_{j-1}<y^{*}<k_{j}then\;y=j,\;\;\;for\;j=2\;,\;3\;\;and$$

$$If\;k_{3}<y^{*}{\;}_{\;}then\;y=4$$

where y* is a latent variable [19]. The variable, y* represents the respondent’s agreement to compare against the thresholds. For each survey respondent *i*, y* can be explained by a set of variables *X*_*i*_,

$$\;y^{*}=\;\beta X_{i}+\;u_{i}{}_{\;}.$$

The probability of each rank outcome *j* can then be estimated

$$Pr\left(y_{i}=j\right)=Pr(k_{j-1}<\;\beta X_{i}+\;u_{i}<\;k_{i})$$

depending on the outcome of the regression falling between *k*_*j*_ and *k*_*j-1*_ [19]. The coefficients, while representing the direction of change, cannot be interpreted in terms of magnitude of change, so marginal effects were estimated [19]. The regressions were performed using STATA statistical software [22].

All explanatory variables employed in the models were discrete binary variables based on the demographic information provided by respondents. Being male *(Male*) and having a college degree (*Degree*) were represented as dummy variables with (1) indicating that this demographic was present. Age and income were each represented by two binary variables, *Age18 to 34* and *Age55 to 88*, and *Inclow* (less than $50,000 in annual household income) and *Inchigh*: ($75,001 or more household income), respectively (with bases, or omitted categories of Age 35 to 54, and middle income $50,001 to $75,000). Geographic region of residence for respondents was represented in the model through three binary variables, namely *Northeast*, *South*, and *Midwest* (*West* served at the base or omitted region). *Zika* was a dummy variable with (1) representing an affirmative answer to the question *Were you aware of the current Zika virus outbreak before participating in this survey*? Similarly, *GM* was a dummy variable with (1) representing a response of yes to the question *Were you aware that a biotechnology company has developed genetically modified mosquitoes (specifically the Aedes Aegypti mosquito) which produce offspring that do not survive to adulthood when they mate*?

### Logit models

The nature of the debate surrounding GM uses and the rather discrete paths forward surrounding GM uses, of either allow or not allow usage, make a discrete response surrounding GM easily/directly interpretable. In order to facilitate interpretation of responses with regard to acceptance of GM uses, the responses were discretized into the general categories of agree and disagree. For each of the five uses a discrete dependent variable was created with (0) being the combined “strongly disagree” and “disagree” to form “strongly/disagree” and (1) being the combined “strongly agree” and “agree” to form “strongly/agree.” In the logit models employed, the probability of respondent(s) agreeing with the use of GM technology for a particular category can be estimated and is represented by

$$Pr_{i}(y=1|X)=\frac{e^{\beta X_{i}}}{1+e^{\beta X_{i}}}$$

where *i* is the individual and X_i_ is the vector of variables and β is a vector of coefficients [18]. The coefficients, while representing the direction of change, cannot be interpreted in terms of magnitude of change [19], so marginal effects were estimated.

## Results

### Respondent demographics

A total of 964 completed responses were collected and a summary of the demographics of respondents can be found in Table 1. While the proportions of the U.S. population were targeted, and the survey was conducted with quotas in place, researchers acknowledge deviations exist and do not assert the sample to be perfectly representative of the population. Of the total respondents 47% reported being male. Twenty-eight percent of respondents were in the age range 18 to 34, 38% 35 to 54, and the remaining 34% were 55 to 88 years of age. Twenty-three percent of the sample selected the Midwest as a place of residence, 35% the South, 23% the West, and 19% the Northeast. Income was divided into three categories. Forty-eight percent of the sample reported an annual household income of less than $50,000 and 33% of the sample reported annual household income of $75,001 or more. The majority of the sample (59%) reported possessing a college degree. Recent travel data was collected from respondents, in particular travel to the Caribbean where the ZIKV outbreak was most active during the data collection period. Twenty-five percent of the sample reported they had traveled to the Caribbean in the two years preceding their participation in the survey.

**Table 1:** Summary statistics for respondent demographics and travel experience (n = 964).

Variable Description	% of Respondents	U.S. Census Bureau Estimates (2010 Census; Revised 2014)
**Gender**		
Male	47	51
Female	53	49
**Age**		
18 to 34	28	31
35 to 54	38	36
55 to 88	34	33
**Region**		
Midwest	23	38
South	35	22
West	23	22
Northeast	19	18
**Income**		
Less than $50,000	48	20
$50,001-$75,000	19	18
$75,001or more	33	32
**Education**		
Has not obtained a College degree or the equivalent	41	57
Has obtained a College degree or the equivalent	59	43
**Have you or a member of your household visited the Caribbean (for any reason) in the past 2 years**		
Yes, an adult member of my household (including myself) has visited in the past 24 months	25	

### Respondent acceptance of GM uses

**Fig 1:**
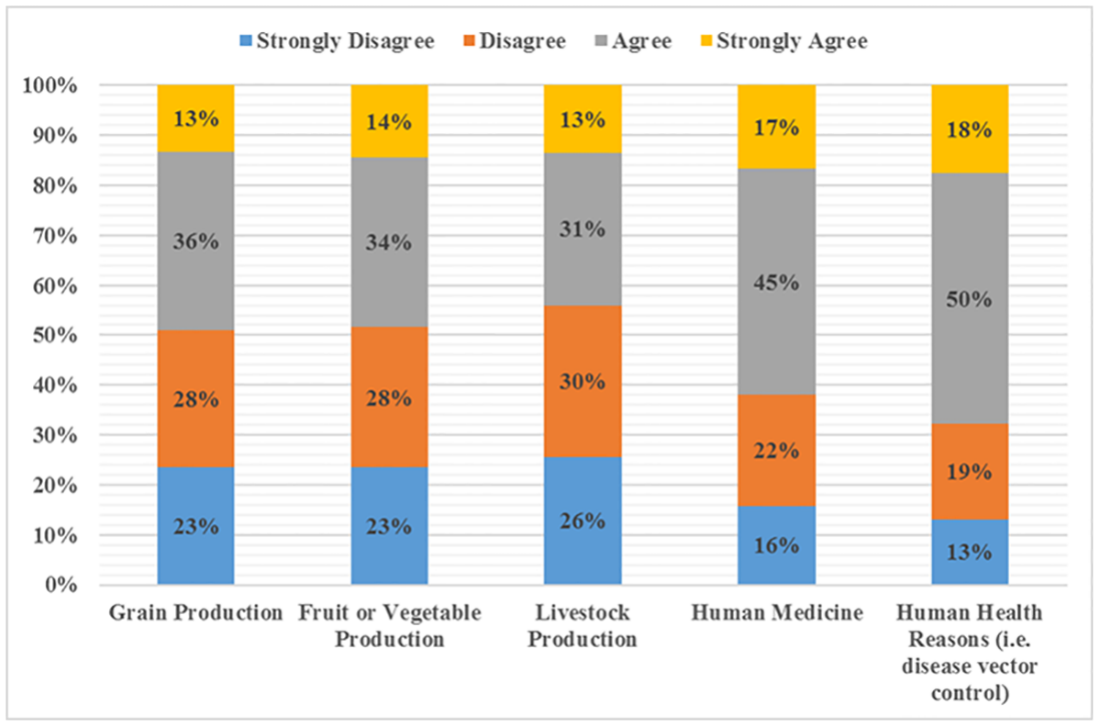
Summary of sample acceptance of GMOs across five categories/uses (n = 964).

Fig 1 summarizes respondent’s agreement with five uses of GM technology. Aggregating “agree” and “strongly agree” into a single category (representing general agreement with the use or acceptance of the technology) revealed less than half (44%) of the sample accepted the use of GM in livestock production. Grain production and fruit and vegetable production showed similar levels of agreement making up 49% and 48% of responses, respectively. Table 2 displays p-values for difference in the proportions of the sample accepting one GM use versus another. The proportion of respondents who agreed with GM for grain production was not statistically different from those who agreed with GM for fruit or vegetable production. The proportion of those who agreed with GM use in livestock production was significantly different from the proportion who agreed with grain production and fruit or vegetable production (at the 5% and 10% levels). The two categories with the highest levels of acceptance for GM use were human medicine and human health reasons with 62% of and 68% of respondents in agreement. Notably, the proportions agreeing with the use of GM for human medicine and human health reasons were statistically different from all other categories.

**Table 2:** P-values from the Fisher’s Exact test to determine if proportion of sample in agreement with acceptability of GM is statistically different between uses.

	Grain Production	Fruit or Vegetable Production	Livestock Production	Human Medicine	Human Health Reasons
Grain Production					
Fruit or Vegetable Production	.8198				
Livestock Production	.0319	.0551			
Human Medicine	.0000	.0000	.0000		
Human Health Reasons	.0000	.0000	.0000	.0087	

Interpretation is such that a p-value of less than 0.05 indicates statistically significant differences in proportions at the 5% level.

Pairwise correlations estimate the direction, significance, and strength of relationships between variables [19]. Pairwise correlations investigating relationships between acceptance of GM across the uses studied are presented in Table 3. It was found that increasing agreement with any one use was positively and statistically related to agreement with any other use. The strongest relationship was between use in grain production and for fruit or vegetable production (a coefficient of .8894), the weakest relationship was between agreement with use for human health reasons and for livestock production (a coefficient of .5821).

**Table 3:** Pairwise correlations between GM uses.

	Grain Production	Fruit or Vegetable Production	Livestock Production	Human Medicine
Fruit or Vegetable Production	0.8894***			
Livestock Production	0.8204***	0.8250***		
Human Medicine	0.6798***	0.6695***	0.6677***	
Human Health Reasons	0.6140***	0.6027***	0.5821***	0.7418***

***p < .00

Key demographic proportions of respondents who reported acceptance of the five different uses or categories of GM are displayed in Table 4 and statistical differences between the groups are marked with differing letters. Males more often reported agreement with all five categories than did females. The majority of male respondents reported agreement with all five categories, with the largest agreement seen for human medicine (70.1% of males) and human health reasons (72.6% of males). In contrast, females more often disagreed with GM uses, with the exceptions of human medicine and human health reasons. Human medicine and human health reasons were the only two uses for which a majority of females reported agreement with the use of GM.

**Table 4:** Cross tabulations between respondent demographics and acceptance of genetically modified organisms across studied categories/uses (n = 964).

	Gender		Age			Region				Income			Education	
	Male	Female	18 to 34	35 to 54	55 to 88	Northeast	South	Midwest	West	Less than $50,000	$50,001 -$75,000	$75,001 or more	No College degree or the equivalent	College degree or the equivalent
**Grain Production**														
Strongly/ Disagree	40.7a	60.2b	44.1a	49.9a	58.0b	49.4a	50.6a	55.6a	48.4a	60.7a	50.0b	37.7c	58.6a	45.8b
Strongly/ Agree	59.3a	39.8b	55.9a	49.9a	42.0b	50.6a	49.4a	44.4a	51.6a	39.3a	50.0b	62.3c	41.4a	54.2b
**Fruit or Vegetable Production**														
Strongly/ Disagree	40.0a	61.7b	45.9a	49.3a	58.6b	51.1a	51.2a	55.2a	48.9a	61.6a	48.9b	38.6c	59.6a	46.0b
Strongly/ Agree	60.0a	38.3b	54.1a	50.7a	41.4b	48.9a	48.8a	44.8a	51.1a	38.4a	51.1b	61.4c-	40.4a	54.0b
**Livestock Production**														
Strongly/ Disagree	45.1a	65.4b	49.3a	54.3a	63.1b	54.4a	56.1a	59.6a	53.0a	63.7a	56.1a	44.5b	62.7a	51.2b
Strongly/ Agree	54.9a	34.6b	50.7a	45.7a	36.9b	45.6a	43.9a	40.4a	47.0a	36.3a	43.9a	55.5b	37.3a	48.8b
**Human Medicine**														
Strongly/ Disagree	29.9a	45.1b	31.9a	38.0ab	42.9b	35.0a	37.4a	41.7a	37.4a	45.1a	35.0b	29.3b	47.0a	31.8b
Strongly/ Agree	70.1a	54.9b	68.1a	62.0ab	57.1b	65.0a	62.6a	58.3a	62.6a	54.9a	65.0b	70.7b	53.0a	68.2b
**Human Health Reasons (i.e. disease vector control)**														
Strongly/ Disagree	27.4a	36.5b	32.2a	32.8a	31.7a	33.9a	31.3a	35.0a	29.7a	39.1a	30.6b	23.4b	39.1a	27.5b
Strongly/ Agree	72.6a	63.5b	67.8a	67.2a	68.3a	66.1a	68.7a	65.0a	70.3a	60.9a	69.4b	76.6b	60.9a	72.5b

Statistically, like letters (I.e. a, b, c) are not different from themselves but are different from each other. For the cross tab analysis the 4-category responses were condensed into two-category responses. “Strongly agree” and “Agree” become “Strongly / agree” and “Strongly Disagree” and “Disagree” become” Strongly/ Disagree”.

Across age brackets analyzed, younger respondents tended to agree more with the use of GMO for all categories studied, except for the use of GM for human health reasons, for which there were not any significant difference amongst age groups. There were not any statistically significant differences seen across respondents from the four regions of the U.S. studied for any of the five GM uses investigated.

Statistically significant differences in rates of GM acceptance were seen across the three income brackets studied for all five GM uses studied. In general, the lowest income group more often reported disagreement with the use of GM for grain production, fruit and vegetable production, and livestock production than did higher income groups. The highest income group had greater than 50% of respondents in agreement with each use of GM studied and greater than 70% agreement for the human medicine and human health categories. Similar to differences across income levels, the acceptance of GM between respondents with and without college degrees differed for every one of the uses studied. For each use studied, a larger proportion of respondents with a college degree reported agreement with the use of GM than respondents without a college degree. For each use, except livestock production, greater than 50% of respondents with a college degree reported acceptance of the use of GM. In contrast, only human medicine and human health reasons saw greater than 50% acceptance of the use of GM for respondents without a college degree.

Five individual ordered and binary logit models (with associated marginal effects) were estimated in order to explain the acceptance of GM across five different categories or uses (grain production, fruit or vegetable production, livestock production, human medicine, and human health reasons) and are displayed in Table 5 (ordered logit coefficient estimates), 6 and 7 (ordered logit marginal effects), and 8 (binary logit coefficient estimates and marginal effects). A number of variables behaved similarly across all five logit models estimated. For example, all region of residence variables were insignificant across all five logit models. Given the lack of significant differences across regions in the cross tabulations (Table 4), this lack of significance in the logit models is not surprising. In addition to region, all age variables showed no significance in all five models. Awareness of ZIKV was an insignificant variable in all five models.

**Table 5:** Ordered logit coefficient (SE) results for acceptance of each of the five genetically modified organism categories/uses (n = 964).

Variable	Grain Production	Fruit or Vegetable Production	Livestock Production	Human Medicine	Human Health Reasons
Male	.7520*** (.1231)	.7659*** (.1231)	.7199*** (.1227)	.5882*** (.1247)	.3809*** (.1255)
Age18 to 34	.2448 (.1494)	.2008 (.1479)	.1883 (.1486)	.1897 (.1519)	.0523 (.1535)
Age55 to 88	-.2657* (.1428)	-.2859* (.1430)	-.2539* (.1422)	-.1486 (.1451)	-.0098 (.1464)
Northeast	-.0220 (.1875)	-.0666 (.1862)	-.0983 (.1859)	.1529 (.1878)	-.1205 (.1905)
South	.1037 (.1622)	.1137 (.1614)	.0786 (.1633)	.1573 (.1638)	.0775 (.1659)
Midwest	-.0385 (.1772)	-.0826 (.1768)	-.0997 (.1783)	-.0253 (.1782)	-.0570 (.1813)
Inclow: Less than $50,000	-.1639 (.1650)	-.2351 (.1641)	-.0807 (.1654)	-.2360 (.1693)	-.0628 (.1702)
Inchigh: $75,001or more	.5419*** (.1738)	.4823*** (.1724)	.4769*** (.1736)	.1828 (.1760)	.4875*** (.1781)
Degree: College degree or the equivalent	.1375 (.1308)	.1485 (.1304)	.1892 (.1311)	.3960*** (.1336)	.2472** (.1346)
Zika: Zika awarness	.0226 (.1646)	-.1195 (.1657)	-.1771 (.1671)	-.1264 (.1704)	-.0543 (.1725)
GM:GM awareness	.3958*** (.1441)	.3348** (.1422)	.5568*** (.1438)	.6581*** (.1470)	.6011*** (.1490)
*Cut1*	*-*.*0220* *(*.*1875)*	-.*8391* *(*.*2495)*	-.*6469* *(*.*2508)*	*-1*.*1975* *(*.*2588)*	*-1*.*4063* *(*.*2632)*
*Cut2*	.*1037* *(*.*1622)*	.*5072* *(*.*2477)*	.*7535* *(*.*2508)*	.*0626* *(*.*2535)*	-.*2142* *(*.*2556)*
*Cut3*	-.*0385* *(*.*1772)*	*2*.*3845* *(*.*2610)*	*2*.*526245* *(*.*2645)*	*2*.*3491* *(*.*2666)*	*2*.*2055* *(*.*2672)*
*Log Likelihood* *Pseudo R2* *Prob> Chi2*	*-1228*.*8397* *0*.*0424* *0*.*0000*	*-1240*.*2618* *0*.*0419* *0*.*0000*	*-1241*.*0178* *0*.*0416* *0*.*0000*	*-1184*.*9538* *0*.*0416* *0*.*0000*	*-1156*.*5959* *0*.*0285* *0*.*0000*

For all variables the marginal effect is for discrete change of dummy variable from 0 to 1

Bolded values indicate statistical significance;

p-values:

* p < .10,

** p < .05,

***p < .00

The marginal contributions for the ordered logits are presented in Tables 6 and 7; for example, the marginal contribution of being male to the acceptance of grain production is as follows, males are 12% less likely to strongly disagree, 6% less likely to disagree, 10% more likely to agree, and 7% more likely to strongly agree. Two variables were significant in all models, namely, being male and GM awareness. Being male was significant and positive for all models, meaning that respondents who reported being male were more likely to agree with all five of the uses of GM studied than those who were female. In the binary models, Table 8, the marginal contribution of being male for each model ranged from 0.069 to 0.209, with the lowest probability belonging to human health reasons. For brevity, only the marginal contribution of the binary logits will be discussed. The only other coefficient estimate that was significant across all models was GM awareness, and it was positive in all five models. In this analysis, GM awareness was incorporated as those who self-reported awareness of GM mosquito technology. Those who were reportedly aware of GM mosquito technology were also more likely to agree with all uses of GM technology investigated. The marginal contribution in the binary models ranges from 0.100 to 0.147, with the lowest increase in probability being for grain production and the highest for livestock production. Given the limited measurement of GM awareness in this analysis, future work with more breadth of analysis regarding various aspects of GM awareness are warranted.

**Table 6:** Marginal effects of ordered logits for food oroduction categories/uses.

	Grain Production				Fruit or Vegetable Production				Livestock Production			
Variable	Outcome 1	Outcome 2	Outcome 3	Outcome 4	Outcome 1	Outcome 2	Outcome 3	Outcome 4	Outcome 1	Outcome 2	Outcome 3	Outcome 4
Male	-.1252*** (.0205)	-.0605*** (.0114)	.1065*** (.0181)	.0793*** (.0139)	-.1276*** (.0204)	-.0614*** (.0115)	.1022*** (.0172)	.0868*** (.0148)	-.1281*** (.0216)	-.0481*** (.0102)	.0987*** (.0174)	.0774*** (.0140)
Age18 to 34	-.0400* (.0237)	-.0210 (.0136)	.0348* (.0206)	.0262 (.0167)	-.0330 (.0238)	-.0170 (.0132)	.0272 (.0195)	.0229 (.0175)	-.0332 (.0256)	-.0134 (.0113)	.0262 (.0203)	.0204 (.0166)
Age55 to 88	.045* (.0252)	.0203* (.0105)	-.0397* (.0218)	-.0264* (.0138)	.0495* (.0253)	.0216** (.0104)	-.0405* (.0207)	-.0306** (.0149)	.0467* (.0266)	.0155* (.0082)	-.0364* (.0206)	-.0258* (.0141)
Northeast	.0037 (.0319)	.0017 (.0149)	-.0032 (.0278)	-.0022 (.0190)	.0113 (.0322)	.0052 (.0142)	-.0093 (.0265)	-.0072 (.0199)	.0180 (.0346)	.0061 (.0109)	-.0140 (.0269)	-.0100 (.0186)
South	-.0173 (.0269)	-.0085 (.0136)	.0151 (.0235)	.0107 (.0170)	-.0190 (.0267)	-.0093 (.0135)	.0156 (.0220)	.0127 (.0183)	-.0141 (.0291)	-.0053 (.0112)	.0111 (.0229)	.0083 (.0174)
Midwest	.0065 (.0302)	.0030 (.0140)	-.0057 (.0263)	-.0039 (.0179)	.0141 (.0306)	.0064 (.0134)	-.0116 (.0251)	-.0089 (.0189)	.0182 (.0330)	.0062 (.0106)	-.0142 (.0257)	-.0102 (.018)
Inclow: Less than $50,000	.0277 (.0279)	.0132 (.0133)	-.0241 (.0243)	-.0168 (.0169)	.0398 (.0279)	.0188 (.0131)	-.0327 (.0229)	-.0259 (.0181)	.0145 (.0298)	.0053 (.0109)	-.0114 (.0234)	-.0084 (.0173)
Inchigh: $75,001or more	-.0868*** (.0264)	-.0477*** (.0168)	.0746*** (.0226)	.0598*** (.0208)	-.0777*** (.0265)	-.0421** (.0165)	.0632*** (.0214)	.0567*** (.0217)	-.0823*** (.0287)	-.0356** (.0147)	.0646*** (.0226)	.0533** (.0208)
Degree: College degree or the equivalent	-.0233 (.0224)	-.0109 (.0103)	.0203 (.0195)	.0139 (.0132)	-.0252 (.0223)	-.0117 (.0102)	.0208 (.0184)	.0162 (.0141)	-.0344 (.0241)	-.0121 (.0082)	.0269 (.0188)	.0196 (.0134)
Zika: Zika awarness	-.0038 (.0280)	-.0018 (.0131)	.0033 (.0244)	.0023 (.0167)	.0197 (.0267)	.0101 (.0147)	-.0162 (.0219)	-.0136 (.0194)	.0309 (.0282)	.0129 (.0134)	-.0244 (.0224)	-.0194 (.0192)
GM:GM awareness	-.0634*** (.0218)	-.0350** (.0141)	.0549*** (.0190)	.0435** (.0171)	-.0541** (.022)	-.0293** (.0136)	.0442** (.0180)	.0392** (.0176)	-.0937*** (.0225)	-.0441*** (.0139)	.0734*** (.0180)	.0644*** (.0185)

For all variables the marginal effect is for discrete change of dummy variable from 0 to 1

Bolded values indicate statistical significance;

p-values:

* p < .10,

** p < .05,

***p < .01

**Table 7:** Marginal effects of ordered logits for health categories/uses.

	Human Medicine				Human Health Reasons			
Variable	Outcome 1	Outcome 2	Outcome 3	Outcome 4	Outcome 1	Outcome 2	Outcome 3	Outcome 4
Male	-.0704*** (.0151)	-.0646*** (.0141)	.0588*** (.0135)	.0761*** (.0166)	-.0401*** (.0133)	-.0410*** (.0136)	.0283*** (.0100)	.0528*** (.0176)
Age18 to 34	-.0222 (.0173)	-.0212 (.0171)	.0185 (.0140)	.0248 (.0205)	-.0055 (.0160)	-.0056 (.0166)	.0039 (.0112)	.0072 (.0214)
Age55 to 88	.0182 (.0181)	.0164 (.0159)	-.0160 (.0161)	-.0186 (.0179)	.0010 (.0155)	.0010 (.0158)	-.0007 (.0113)	-.0013 (.0200)
Northeast	-.0178 (.0211)	-.0171 (.0212)	.0148 (.0168)	.0201 (.0255)	.0131 (.0214)	.0130 (.0205)	-.0100 (.0171)	-.0161 (.0248)
South	-.0186 (.0191)	-.0175 (.0183)	.0158 (.0160)	.0203 (.0215)	-.0081 (.0173)	-.0083 (.0179)	.0058 (.0121)	.0107 (.0231)
Midwest	.0030 (.0217)	.0028 (.0197)	-.0026 (.019)	-.0032 (.0224)	.0061 (.0197)	.0061 (.0196)	-.0045 (.0149)	-.0077 (.0244)
Inclow: Less than $50,000	.0286 (.0206)	.0261 (.0187)	-.0247 (.0180)	-.0299 (.0214)	.0066 (.0181)	.0068 (.0184)	-.0048 (.0132)	-.0086 (.0233)
Inchigh: $75,001or more	-.0216 (.0204)	-.0204 (.0197)	.0182 (.0168)	.0238 (.0234)	-.0487*** (.0170)	-.0521*** (.0189)	.0302*** (.01)	.0707** (.0273)
Degree: College degree or the equivalent	-.0491*** (.0171)	-.0433*** (.0146)	.0432*** (.0157)	.0492*** (.0163)	-.0267* (.0148)	-.0267* (.0146)	.0199* (.0116)	.0334* (.0179)
Zika: Zika awarness	.0148 (.0193)	.0141 (.0192)	-.0123 (.0156)	-.0165 (.0230)	.0056 (.0178)	.0058 (.0186)	-.0040 (.0122)	-.0075 (.0242)
GM:GM awareness	-.0717*** (.0148)	-.0733*** (.0167)	.0521*** (.0105)	.0929*** (.0230)	-.0578*** (.0133)	-.0637*** (.0157)	.0312*** (.0079)	.0903*** (.0244)

For all variables the marginal effect is for discrete change of dummy variable from 0 to 1

Bolded values indicate statistical significance;

p-values:

* p < .10,

** p < .05,

***p < .01

**Table 8:** Logit regression results for binary acceptance of each of the five genetically modified organism categories/uses (n = 964).

	Grain Production		Fruit or Vegetable Production		Livestock Production		Human Medicine		Human Health Reasons (i.e. disease vector control)	
Variable	Coefficient (SE)	Marginal effect (SE)	Coefficient (SE)	Marginal effect (SE)	Coefficient (SE)	Marginal effect (SE)	Coefficient (SE)	Marginal effect (SE)	Coefficient (SE)	Marginal effect (SE)
Male	.7661*** (.1396)	**.1892** **(.03365)**	.8505*** (.1401)	**.2094** **(.0335)**	.7853*** (.1401)	**.1914** **(.0334)**	.5595*** (.1425)	**.1288** **(.0322)**	.3233** (.1460)	**.0691** **(.0309)**
Age18 to 34	.2087 (.1717)	.0521 (.0428)	.0958 (.1724)	.0239 (.0430)	.1431 (.1713)	.0353 (.0424)	.2459 (.1782)	.0563 (.0400)	-.0123 (.1788)	-.0026 (.0385)
Age55 to 88	-.2445 (.1634)	-.0609 (.0406)	-.2501 (.1643)	-.0622 (.0407)	-.2716 (.1657)	-.0663 (.0401)	-.0726 (.1644)	-.0169 (.0384)	.1706 (.1710)	.0362 (.0359)
Northeast	-.1477 (.2130)	-.0368 (.053)	-.2136 (.2143)	-.0531 (.0529)	-.1634 (.2139)	-.0399 (.0517)	.0757 (.2192)	.0175 (.0503)	-.2341 (.2225)	-.0516 (.0502)
South	-.0616 (.1863)	-.0154 (.0465)	-.0743 (.1872)	-.0185 (.0467)	-.1380 (.1872)	-.0338 (.0457)	.0830 (.1901)	.0192 (.0439)	.0094 (.1970)	.0020 (.0422)
Midwest	-.2130 (.2026)	-.0530 (.0502)	-.1934 (.2035)	-.0481 (.0504)	-.2079 (.2041)	-.0507 (.0492)	-.0592 (.2045)	-.0138 (.0479)	-.1367 (.2115)	-.0297 (.0466)
Inclow: Less than $50,000	-.2521 (.1879)	-.0629 (.0467)	-.3495* (.1890)	**-.0870** **(.0468)**	-.1112 (.1914)	-.0273 (.0470)	-.2350 (.1926)	-.0546 (.0447)	-.2766 (.1961)	-.0594 (.0421)
Inchigh: $75,001or more	.5506*** (.1975)	**.1366** **(.0483)**	.4718** (.1981)	**.1174** **(.0488)**	.5133*** (.1981)	**.1267** **(.0487)**	.2129 (.2080)	.0490 (.0473)	.3219 (.2144)	.0676 (.0439)
Degree: College degree or the equivalent	.1440 (.1500)	.0359 (.0374)	.2046 (.1509)	.0510 (.0375)	.1321 (.1524)	.0324 (.0373)	.4298*** (.1505)	**.1006** **(.0353)**	.2811* (.1544)	**.0609** **(.0336)**
Zika: Zika awarness	.0075 (.1930)	.0018 (.0482)	-.2514 (.1940)	-.0627 (.0482)	-.1963 (.1936)	-.0486 (.0481)	-.2526 (.1970)	-.0573 (.0434)	-.1339 (.1980)	-.0282 (.0410)
GM:GM awareness	.4023** (.1609)	**.1001** **(.0397)**	.4085** (.1615)	**.1017** **(.0398)**	.5969*** (.1597)	**.1476** **(.0392)**	.6179*** (.1720)	**.1371** **(.0359)**	.5813*** (.1792)	**.1181** **(.0339)**
Constant	-.5359 (.2848)		-.3029 (.2851)		-.6362 (.2870)		.0335 (.2898)		.4654 (.2960)	
Log Likelihood Pseudo R2 Prob> Chi2	-620.096 .0717 .0000		-615.349 .0784 .0000		-613.661 .0772 .0000		-602.758 .0582 .0000		-581.565 .0406 .0000	

For all variables the marginal effect is for discrete change of dummy variable from 0 to 1

Bolded values indicate statistical significance;

p-values:

* p < .10,

** p < .05,

***p < .01

When estimating acceptance of grain production, fruit and vegetable production, and livestock production the highest income category was significant and positive, indicating that those with higher incomes were more likely to be in agreement with these GM uses than those in the middle income category. In the ordered logit models (although not in the binary logit models) the highest income category was also positive and significant for acceptance of GM use for human health reasons. In the binary logit models for fruit or vegetable production the lower income category was also significant (negative), which suggests that those in the lower income category were less likely to agree with GM use than the middle income. The positive and significant coefficients for having a college degree for both human medicine and human health uses of GM indicate that those with higher education were more likely to agree with GM use for these reasons than those without a college degree.

### ZIKV outbreak awareness and mosquito control preferences

Respondents were asked about their awareness of ZIKV at the time of participating in the survey. Only 8.9% were unaware of any outbreak while 83.6% were aware of the ZIKV outbreak and 7.5% were aware of an active outbreak but could not recall the name of the illness. In total, 81.2% of respondents were aware of the (much publicized) impacts of ZIKV on pregnant women. Widmar et al. [23] explored respondents’ knowledge of ZIKV, mosquito prevention measures, and travel intentions in detail.

Respondents were asked three distinct questions linking human illness and mosquitoes. A total of 40.9% of respondents were aware that ZIKV was spread by the same type of mosquito that spreads Dengue and Chikungunya, while only 34.4% were aware the mosquito that spreads ZIKV is primarily a daytime biting mosquito [23]. When asked about what mosquito bite prevention respondent’s used, 49.5% of respondents indicated that they or another household member used bug spray or repellent, 31.2% indicated they or others used clothing (long sleeves or pants) for the specific purpose of insect bite control, 21.9% reported active management of standing water around the home by a household member, 7.4% reported the use of mosquito nets by their household, and 13.5% reported household use of insect sprays, foggers, or other products to control mosquitoes in the house or yard [23]. In total 41.1% of respondents reported that nobody in their household actively managed mosquito bite prevention [23].

Respondents were asked if they were aware of the development of a GM mosquito (Aedes Aegypti) which mates to bear terminal offspring and the majority (72.5%) responded with no. In addition to awareness about the potential GM solution to combat ZIKV, respondents were asked about their willingness to support the release of the GM mosquitoes in both the Caribbean and the U.S. to reduce illnesses and deaths from mosquito-borne diseases. Overall, over three-quarters of respondents were supportive of introducing the GM mosquitoes in both locales, with 75.2% in support for the Caribbean and 78% in support for the U.S.

Those who said they would not support the introduction of the GM mosquitoes were asked a follow up question and were provided a number of options, of which, they were allowed to select more than one. For the U.S. (Caribbean) 15.6% (14.6%) indicated that they would not support introduction of the GM mosquito under any circumstances, 4.8% (6.6%) indicated that they would support it if birth defect and death rates increased beyond what has already been observed, 2.4% (3.7%) said they would support it if adult death rates increased beyond what has already been observed, and 1.3% (2.0%) indicated that there was some other circumstance in which they would support the use of the technology.

Respondents were asked to indicate between most preferred, neutral, or least preferred method for three methods of potential mosquito control in the U.S. and Caribbean, specifically fogging and pesticide spraying in public places, release of GM mosquitoes to reduce populations, and personal use of insect repellents/bug sprays and protective clothing. Respondents were distributed nearly in thirds for all levels of preference for all methods, for both the U.S. and the Caribbean. For the Caribbean the largest percentage (36%) of respondents found fogging and pesticide spraying in public places to be the least preferred method. The remainder of control methods were preferred in each of the three categories at nearly an identical rate to all others (ranging from 31% to 35% for each). For the U.S. the biggest proportion of respondents in a single category was the 37% in the least preferred category for fogging and pesticide spraying in public places. The release of GM mosquitoes to reduce populations was reported by 37% of respondents in the neutral category, and personal use of insect repellents/bug sprays and protective clothing was rated most preferred by 38% of respondents.

### Cross tabulations of mosquito control mechanisms across demographics

Acceptance of various forms of mosquito control, including the use of a GM mosquito, was analyzed across various respondent demographics. Table 9 displays the percentage of respondents within various demographic groups who were aware of the GM mosquito, would support the release of the mosquito, and who preferred various mosquito control techniques for use in the Caribbean and U.S. Males and females reported awareness of the GM mosquito at different rates, with 35.4% of males having been aware, while only 20.5% of females were aware. A higher percentage of the youngest age category (18 to 34 years of age) reported awareness of the GM mosquito, with 34.8% of respondents compared to 26.5% and 22.7% in the two older categories. The percent of respondents who were aware of the GM mosquito was higher amongst college degree holders (34.0%) than non-degree holders (18.0%) and the two higher income categories reported higher awareness than the lowest income category studied.

**Table 9:** Cross tabulations between respondent demographics and preference for mosquito control (including the use of genetically modified mosquitoes) (n = 964).

	Gender		Age			Region				Income			Education	
	Male	Female	18 to 34	35 to 54	55 to 88	Northeast	South	Midwest	West	Less than $50,000	$50,001 -$75,000	$75,001or more	No College degree or the equivalent	College degree or the equivalent
**Were you aware that a biotechnology company has developed genetically modified mosquitoes (specifically the Aedes Aegypti mosquito) which produce offspring that do not survive to adulthood when they mate?**^1^														
Yes	35.4a	20.5b	34.8a	26.4b	22.7b	29.4b	27.8ab	20.6a	32.4b	18.8a	31.1b	38.0b	18.0a	34.0b
**Would you support introducing these genetically modified mosquitoes in the Caribbean to reduce illnesses and deaths caused by mosquito-borne diseases?**^1^														
Yes	75.0a	75.4a	75.2a	72.5a	78.2a	75.6a	77.8a	73.5a	73.5a	70.0a	77.8b	81.3b	69.5a	79.1b
**Would you support introducing these genetically modified mosquitoes in the United States to reduce illnesses and deaths caused by mosquito-borne diseases?**^1^														
Yes	77.0a	78.9a	78.1ab	74.9b	81.3a	76.7ab	82.5b	73.1a	77.2ab	73.7a	77.8ab	84.4b	74.9a	80.2a
**Preference for the following mosquito control mechanisms you MOST support for use in the Caribbean**^2^														
**Fogging and pesticide spraying in public places**														
Most preferred	36.1a	26.2b	33.7a	29.2a	30.2a	25.6a	30.4a	33.6a	32.9a	29.8a	31.1a	32.1a	29.7a	31.6a
Least preferred	31.4a	40.8b	35.9a	37.2a	36.0a	37.8a	36.0a	35.9a	36.5a	38.0a	32.8a	36.1a	35.8a	36.8a
**Release of genetically modified mosquitoes to reduce populations**														
Most preferred	33.6a	36.7a	30.0a	31.7a	43.5b	36.1ab	40.0b	32.2ab	29.7a	35.0a	25.6b	41.1a	35.3a	35.3a
Least preferred	30.8a	29.5a	27.8a	32.2a	29.6a	29.4ab	26.0b	35.0a	32.0ab	32.6a	35.6a	23.4b	33.5a	27.7a
**Personal use of insect repellents bug sprays and protective clothing**														
Most preferred	30.3a	37.1b	36.3a	39.1a	26.3b	38.3a	29.2b	34.1ab	37.4a	35.2a	43.3a	26.8b	35.0a	33.2a
Least preferred	37.8a	29.7b	36.3a	30.6a	34.4a	32.8ab	38.0b	29.1a	31.5ab	29.4a	31.7a	40.5b	30.7a	35.4a
**Preference for the following mosquito control mechanisms you MOST support for use in the United States**^2^														
**Fogging and pesticide spraying in public places**														
Most preferred	32.5a	27.3a	31.1a	29.5a	29.0a	27.2a	28.9a	28.7a	34.2a	28.5a	30.3a	21.2a	27.9a	31.1a
Least preferred	33.2a	40.2b	39.6a	36.1a	35.6a	37.2a	36.3a	36.8a	37.9a	36.5a	36.1a	38.0a	34.8a	38.4a
**Release of genetically modified mosquitoes to reduce populations**														
Most preferred	32.7a	32.6a	25.9a	28.9a	42.3b	30.0a	36.0a	33.6a	28.8a	32.4ab	25.0b	37.4a	32.2a	33.0a
Least preferred	31.0a	30.7a	28.9a	32.8a	30.2a	30.6a	28.1a	35.4a	30.6a	33.3a	35.0a	24.9b	34.8a	28.1b
**Personal use of insect repellents bug sprays and protective clothing**														
Most preferred	34.7a	40.0a	43.0a	41.6a	28.7b	42.8a	35.1a	37.7a	37.0a	39.1a	44.4a	31.5b	39.8a	36.0a
Least preferred	35.8a	29.1b	31.5a	31.1a	34.1a	32.2a	35.7a	27.8a	31.5a	30.2a	28.9ab	37.1b	30.5a	33.5a

^1^The ‘no’ responses have been left out of the table and can be obtained by subtracting the percent for ‘yes’ from 100 within the column.

^2^The ‘Neutral’ responses have been left out of the table and can be obtained by subtracting the percent for the sum of the ‘most preferred’ and ‘least preferred’from100 along the column

Support for the introduction of GM mosquitos in the Caribbean differed between college degree holders (79.1% acceptance) and non-degree holders (69.5% acceptance). The two higher income categories studied had higher levels of acceptance than the lowest income bracket analyzed. Support for the introduction of the GM mosquito in the U.S., the highest income category reported 84.4% support which was significantly different from the 73.7% of respondents reporting support in the lowest household income category. The middle income category ($50,001 to $75,000 annual household income) was not significantly different from either the higher or lower category.

Several differences were seen amongst demographics in the stated preferences for mosquito control mechanisms in the U.S. and Caribbean. Females rated fogging and pesticide spraying in public places as least preferred for both the U.S. and Caribbean at a higher rate than males. Males ranked personal use of insect repellant bug sprays and protective clothing as the least preferred method for both the U.S. and Caribbean at a higher rate than females. In both the U.S. and Caribbean a higher percentage of the oldest age group ranked the release of GM mosquitos as most preferred (compared to the other two age brackets). The only significant difference seen across education categories was for the release of GM mosquitoes in the U.S., for which a lower percentage of those with a college degree ranked the release of GM mosquitoes as least preferred.

## Discussion

The results of this study align with past studies that suggest people are more willing to accept the use of GM technology for human medicine and human health reasons (62% and 68% respectively) than for livestock production, grain production, or fruit and vegetable production (44%, 49% and 48% respectively.) Notably, the proportion of survey respondent acceptance of food production uses (grain, fruit and vegetable, and livestock production) differed significantly from the proportion which accepted GM for both human health reasons and human medicine. Perhaps, less obvious in terms of distinction amongst respondents is the difference in acceptance of GM for use in livestock versus grain production (at the 5% level) and fruit or vegetable production (at the 10% level). A potential hypothesis surrounding this difference in acceptance of GM amongst food production uses is the association with animals (livestock) versus plants (crops) and the perceived relationship to human beings. Animals are more human-like than plants and it is conceivable that GM in animals is perceived quite differently than when used in plant production. Regardless of the minor differences in acceptance between livestock and other food production uses, the significant differences, and notably higher acceptance of GM for human medicine and human health reasons, are important results which offer some insight into the potential for accepted GM uses to improve the human condition.

Various willingness-to-pay and willingness-to-accept analyses of consumer preference in various countries have been conducted regarding GM and non-GM food products. Fernandez-Cornejo et al. [24] provide an overview of much of the research done in this area and conclude that while many consumers are willing to pay a premium for non-GM foods, willingness depends on where the study is being performed. Lusk et al. [25] performed a meta-analysis of studies focused on GM vs. non-GM valuation and Europeans appear willing to pay a 42% premium for non-GM over GM, but in Asia the premium is only 16%. Chiang et al. [26] reported that a substantial percentage of consumers across the world believe that GM crops are dangerous for human consumption. This analysis supports previous findings that people have a higher rate of acceptance of GM for human medicine and human health uses than other potential uses (such as food production).

Being male, younger, of higher income, and college educated generally contributed to higher willingness to accept GM technology, which could be related to the access of information. Costa-Font and Mossialos [27] suggested that what they term “dread of GM crops” is at least partially explained by lack of information. Increasing resources, such as income and education, could improve access to information about GM technology, and eventually, understanding. Conversations about GM technology have been rising over time and younger people are avid users of quick information portals, i.e. the internet, and this increased exposure could account for increase GM acceptance.

While, specific awareness of ZIKV was an insignificant variable in all five of the logit models, GM mosquito awareness was statistically significant and positive in contributing to acceptance of all five GM uses. GM mosquito technology could find some support in ZIKV impacted regions and may even be preferred to some other method of control. This finding could suggest information about one aspect of GM technology that could impact the acceptance of another. Thus, promotion of awareness of GM technologies used in health and medicine may be an important component of attempts to gain acceptance (in the realm of public perception) of GM technologies for use in other aspects of improving the human condition (such as through food and nutrition).

Admittedly the measurement of GM awareness in this analysis is limited; specifically only awareness of the GM mosquito was measured (or self-reported) and then used in the models. Additional measures of awareness across the five uses may further inform related analyses. Furthermore, logit models predicting acceptance of GM across five uses employed basic demographics (sex, age, income, region of residence, education) ZIKV awareness, and GM awareness (measured by GM mosquito awareness). Certainly one might consider the potential contribution of factors beyond the scope of this study, including awareness of or experience with GM in medical procedures, occupation, or more specific knowledge on food production. Furthermore, future studies may wish to consider both more specific GM uses, beyond the five broad categories studied here, and perhaps the study of GM acceptance concurrently (rather than each use analyzed separately) to account for within person impacts on responses. The development of a GM mosquito as a means of control for mosquito borne illnesses was a key area of focus around the globe in 2016 relating to human health. Most obviously related in terms of this study were the stated preferences for mosquito control strategies. Respondents were distributed nearly in thirds for all levels of preference for all methods (fogging and spraying in public places, release of GM mosquitoes to reduce populations, and personal use of insect repellents, bug sprays, and protective clothing), for both the U.S. and the Caribbean. In other words, no single method was chosen as the most preferred for any majority of respondents in the sample for either the location. However, for both locations, the largest share of respondents (although not a majority of respondents) found fogging and pesticide spraying to be the least preferred method. It is possible that recent press related to illegal use of pesticides in the U.S. Virgin Islands may have fed fears of public spraying, in particular in resort locations where multiple offenses have been admitted to and families left permanently disabled [28][29]. This finding surrounding acceptability of fogging and spraying leaves room for the further acceptance of GM mosquitoes or GM-derived control methods in the future.

Notably, this study found 72.5% of respondents would support the use of GM mosquitos for illness control in the Caribbean and 78.0% showed support for use in the U.S. Interestingly, region of residence did not significantly explain acceptance of any of the five GM uses investigated. The lack of significance of region is important to consider for GM mosquitos because different regions face different impacts from mosquito populations. Populations in highly impacted regions have expressed interest in preventing the spread of diseases using GM technology. For example, research conducted in Mali showed that most study participants were pragmatic about use of GM mosquitoes as part of the vector control strategy for malaria when they were properly informed about the purposes of the program [30].

Specifically to the U.S. according to a 2016 report, Florida residents favor the use of GM mosquitos 60%, compared to 50% general favor by the remaining U.S. [16]. While not explored here, one reason for the insignificance of region could be national and global coverage of ZIKV in the media, making all more aware, not just those in regions of higher risk. Election day 2016 brought the question of GM mosquito introduction in the U.S. out of questionnaire and survey data collection to the potential for real implementation. While 57% of residents in Monroe County Florida voted in favor of the trail with GM mosquitos, 65% of the 643 residents who voted in Key Haven (the study site) were opposed [31]. Given the nature of the referenda, it is not clear what decisions will result [31], although this recent example from Florida highlights the potential for locale-specific differences, not just regional differences, in acceptance, in particular in those locales targeted for release.

## Conclusions

The objective of this study was to explore U.S. resident’s acceptance of GM across five potential categories or uses, with special focus on GM mosquitos as means of controlling ZIKV spread. A total of 964 responses were collected. Less than half (44%) of the sample accepted the use of GM in livestock production, 49% in grain production, 48% in fruit and vegetable production, 62% in human medicine, and 68% for human health. Statistically significant differences in the proportion of the sample accepting GM uses for human health and human medicine versus GM uses for food production were found. Overall, a significantly higher proportion of respondents were willing to accept GM uses for human medicine and health reasons than for food production (grain, fruits and vegetables, and livestock). Respondents reporting being male, being younger, having higher incomes, and being college educated were more likely to agree with GM technology for any of the five uses. Interestingly, the lower income segment was least likely to support GM uses in agriculture, which may run counter to their own self-interest in that loss of GM varieties would lead to higher food prices, which disproportionally affect the poor.

Specific to ZIKV, 83.6% of respondents were aware of ZIKV, 80.6% were aware that mosquitoes could spread viruses among humans, 72% were not aware of the development of a GM mosquito (Aedes Aegypti); however, 75.2% would support the use of GM mosquito technology for use in the Caribbean and 78.0% for use in the U.S. Generally, males, younger respondents, college degree holders, and those with higher incomes were more likely to be aware of the development of a GM mosquito.

Several limitations to this study exist, including the potential for overstating acceptance of GM uses impacting human health, and in particular for disease vector control, given the incorporation of significant focus on ZIKV and control mechanisms. As with any survey analysis, one must consider the specific wording of questions and context in which they were asked. Certainly questions surrounding ZIKV may have led respondents to have more urgency in addressing human health needs (especially vector control) than they might have otherwise. In this way, the dual-focus of the data collection effort on both GM uses and ZIKV as a case study may have influenced responses. In addition, timing of data collection for this analysis may have influenced responses. Data for this study was collected when ZIKV was heavily featured in media. Admittedly, data collection at any point in time is likely to be influenced by the media or current event happenings of that time. However, additional analyses of GM use acceptance, and perhaps analyses focusing on different aspects of GM awareness (aside from disease vector control) may be advantageous.

These findings provide insight into understanding GM acceptance and the potential impact of GM technology on the human condition. GM mosquitos align with respondent GM acceptance for the uses of human medicine and human health which could benefit regions highly impacted by ZIKV as the GM mosquito technology is used and supported to control the spread of the virus. Future studies could consider the overlap of GM food and GM health technology (such as Golden Rice, a genetically engineered rice cultivar that produces vitamin A). Furthermore, future studies should consider acceptance of GM uses concurrently, rather than independently; studying GM uses concurrently may involve study of more than the five uses analyzed here, likely with more specific GM uses focused upon to facilitate study of specific processes or uses of the technology.

## Ethics statement

The survey utilized in this study was approved by the Purdue University Social Sciences Institutional Review Board (IRB) Human Research Protection Program (IRB Protocol Number 1602017146).

## Supporting information

S1 File Data. (XLSX)

S2 File Word document survey instrument. (DOCX)
