# Supplementary material for: When is genetic modification socially acceptable? When used to advance human health through avenues other than food
Source: PLoS One. 2017 Jun 7;12(6):e0178227. doi: 10.1371/journal.pone.0178227 (PMC5462347; doi:10.1371/journal.pone.0178227)
Supplement: S2 File — (DOCX) [file pone.0178227.s002.docx]

February 2016

**Dear US resident,**

Researchers at Purdue University are conducting a survey of US residents in an effort to examine and understand your concerns about the Zika virus and your preferences for various control methods.

This applied research and Extension effort has been approved by Purdue’s Institutional Review Board.  Your participation is strictly voluntary and your response to this survey will be kept completely anonymous. You must be 18 years of age or older in order to participate in this study.

Please identify your age:

- 18 to 24 years
- 25 to 34 years
- 35 to 44 years
- 45 to 54 years
- 55 to 64 years
- 65 to 88 years

I am:

- Male
- Female

My region of residence is:

- Northeast (CT, ME, MA, NH, NJ, NY, PA, RI, VT)
- South (AL, AR, DE, DC, FL, GA, KY, LA, MD, MS, NC, OK, SC, TN, TX, VA, WV)
- Midwest (IL, IN, IA, KS, MI, MN, MO, NE, ND, OH, SD, WI)
- West (AK, AZ, CA, CO, HI, ID, MT, NV, NM, OR, UT, WA, WY)

My state of residence (Midwest) is:

- Illinois
- Indiana
- Iowa
- Kansas
- Michigan
- Minnesota
- Missouri
- Nebraska
- North Dakota
- Ohio
- South Dakota
- Wisconsin

My state of residence (South) is:

- Alabama
- Arkansas
- Delaware
- District of Columbia
- Florida
- Georgia
- Kentucky
- Louisiana
- Maryland
- Mississippi
- North Carolina
- Oklahoma
- South Carolina
- Tennessee
- Texas
- Virginia
- West Virginia

My state of residence (West) is:

- Alaska
- Arizona
- California
- Colorado
- Hawaii
- Idaho
- Montana
- Nevada
- New Mexico
- Oregon
- Utah
- Washington
- Wyoming

My state of residence (Northeast) is:

- Connecticut
- Maine
- Massachusetts
- New Hampshire
- New Jersey
- New York
- Pennsylvania
- Rhode Island
- Vermont

My annual pre-tax household income is:

- Less than $25,000
- $25,001 - $50,000
- $50,001-$75,000
- $75,001-$100,000
- $100,001 or more

The best description of my educational background is:

- Did not graduate from high school
- Graduated from high school, Did not attend college
- Attended College, No Degree earned
- Attended College, Associates or Trade Degree earned
- Attended College, Bachelor’s (B.S. or B.A.) Degree earned
- Graduate or Advanced Degree (M.S., Ph.D., Law School)
- Other

Please enter the number of household members - including adults and children - within each age bracket currently living in your household. Please INCLUDE yourself in the count.

|  | Total number of |
| --- | --- |
|  | household members |
| adults (over 18 years) |  |
| children ages 0 to 4 years |  |
| children ages 5 to 10 years |  |
| children ages 11 to 15 years |  |
| children ages 16 to 18 years |  |

Please report the status or intention of pregnancy by any member of your household.  Please select all that apply.

- I am currently pregnant
- Someone else in my household is pregnant.
- There has been a pregnancy in my household in the last 3 years.
- There is an intention to become pregnant by someone in my household in the next 1 year.
- There is an intention to become pregnant by someone in my household in the next 2 - 3 years.
- None of the above/Not applicable to me.

On a scale of one (I do not give any thought to this at all) to five (I think about this constantly), please rank the level of attention or thought you provide to the following aspects of health and well-being. Please select the number on the scale that best represents your level of attention.

|  | 1. I do not give this any thought | 2 | 3 | 4 | 5. I think about this constantly |
| --- | --- | --- | --- | --- | --- |
| Physical fitness |  |  |  |  |  |
| Heart health |  |  |  |  |  |
| Mental health |  |  |  |  |  |
| Cancer |  |  |  |  |  |
| Flu |  |  |  |  |  |
| Antibiotic resistant bacterial infections |  |  |  |  |  |
| Prenatal care |  |  |  |  |  |

What methods of travel (for any reason) have you or any member of your household utilized in the past 12 months?  (Select all that apply.)

- Cruise ship
- Domestic air travel (within the US)
- International air travel (outside the US)
- Private vehicle/car/truck travel (aka "road trip") of greater than 5 hours one-way
- Public bus
- Train
- None of the above

Have you or a member of your household traveled more than 5 hours from your home (for any reason) within the United States in the past 12 months?  (Select all that apply.)

- Yes, I have traveled within the US.
- Yes, an adult member of my household has traveled within the US.
- Yes, a child in my household has traveled within the US.
- No, no adult or child in my household (including myself) has traveled more than 5 hours from our home within the US.

Have you or a member of your household traveled outside the United States (for any reason) the past 12 months?  (Select all that apply.)

- Yes, I have traveled outside the US.
- Yes, an adult member of my household has traveled outside the US.
- Yes, a child in my household has traveled outside the US.
- No, no adult or child in my household (including myself) has traveled outside the US in the past 12 months.

Have you or a member of your household visited the Caribbean (for any reason) in the past 2 years?

- Yes, I have visited in the past 12 months.
- Yes, I have visited 12 - 24 months ago.
- Yes, an adult member of my household has visited in the past 12 months.
- Yes, an adult member of my household has visited 12-24 months ago.
- Yes, a child in my household has visited in the past 12 months.
- Yes, a child in my household has visited 12 - 24 months ago.
- No, no member of my household (including myself) has traveled to the Caribbean in the past 2 years.

Do you and the members of your household take precautions regularly to prevent mosquito bites?  (Select all that apply.)

- Yes, I and/or others in my household use bug spray or insect repellent.
- Yes I and/or others in my household use clothing with long sleeves or pants specifically for insect bite control.
- Yes, I and/or others in my household use mosquito nets or other mechanisms.
- Yes, I and/or others in my household actively remove standing water around our homes.
- Yes, I and/or others in my household use insect sprays, foggers, or other products to control mosquito populations in our yard or home.
- No, nobody in my household actively manages mosquito bite prevention regularly.

The Pan American Health Organization (PAHO) issued an alert regarding the first confirmed Zika virus infection in Brazil in May of 2015.  Several alerts, including travel alerts and warnings, have been issued throughout the Caribbean and in the United States as well. Were you aware of the current Zika virus outbreak before participating in this survey?

- Yes, I am aware of the Zika virus outbreak.
- I recall recently hearing of an outbreak of some kind, but do not recall the name of the illness.
- No, I am not aware of any outbreak.

Are you aware of the potential for microcephaly when pregnant women contract the Zika virus? Microcephaly is a birth defect where a baby’s head is smaller than expected when compared to babies of the same sex and age. Babies with microcephaly often have smaller brains that might not have developed properly.

- Yes, I am aware of this Zika virus impact for pregnant women
- No, I was not aware of this Zika virus impact for pregnant women

Please select all of the details about the Zika virus which you have heard or are familiar with.

- Approximately 1 in 5 people infected with Zika virus will develop Zika and become ill.
- The incubation period (the time from exposure to symptoms) for the Zika virus is not known, but is believed to be a few days to a week.
- The Zika virus illness is usually mild and lasts for several days to a week.
- People sick with the Zika virus do not usually get sick enough to go to the hospital.
- People rarely die of Zika.
- The Center for Disease Control in the United States has recommended that pregnant women postpone travel to destinations where Zika virus transmission is taking place.
- There has been at least one known case of sexual transmission of the Zika virus in the United States.
- I am not familiar with any of these details.

Please select all of the symptoms which you believe are associated with the Zika virus.

- Fever
- Joint pain
- Rash
- Conjunctivitis (red eyes)
- Sneezing
- Muscle pain
- Headache
- None of these symptoms are associated.

Below is an excerpt from an educational campaign by the Center for Disease Control.  Please review this information. (The full information from Center for Disease Control provides information about mosquito bites and prevention can be found at http://www.cdc.gov/chikungunya/pdfs/fs_mosquito_bite_prevention_us.pdf


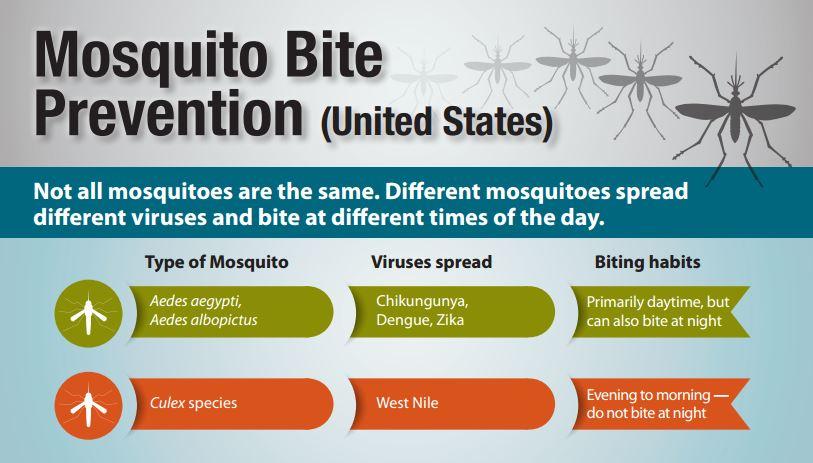


Before taking this survey, were you aware that:

|  | Yes, I was aware of this information | No, I was not aware of this information |
| --- | --- | --- |
| The Zika virus is spread by the same type of mosquitoes as spread Dengue and Chikungunya? |  |  |
| The mosquito that spreads the Zika virus bites primarily during the daytime |  |  |
| Mosquitoes can spread viruses among humans |  |  |

Were you aware that a biotechnology company has developed genetically modified mosquitoes (specifically the Aedes Aegypti mosquito) which produce offspring that do not survive to adulthood when they mate?

- Yes
- No

Scientists have developed a way to modify mosquitoes by adding a gene which produces a protein that stops their cells from functioning normally. The modified mosquitoes die before reaching adulthood. The genetically modified mosquitoes are effectively sterile. When a genetically modified mosquito male mates with a wild female, her offspring will die before reaching adulthood, so the population is reduced. Would you support introducing these genetically modified mosquitoes in the Caribbean to reduce illnesses and deaths caused by mosquito-borne diseases?

- Yes
- No

Can you describe any circumstances under which you WOULD support the introduction of the genetically modified mosquitoes in the Caribbean?  (Select all that apply.)

- No, I do not support the introduction of genetically modified mosquitoes under any circumstances.
- Yes, if birth defect and death rates increased beyond what has already been observed.
- Yes, if adult death rates increased beyond what has already been observed.
- Yes, under other circumstances - Please describe: ____________________

Would you support introducing these genetically modified mosquitoes in the United States to reduce illnesses and deaths caused by mosquito-borne diseases?

- Yes
- No

 Can you describe any circumstances under which you WOULD support the introduction of the genetically modified mosquitoes in the United States?  (Select all that apply.)

- No, I do not support the introduction of genetically modified mosquitoes under any circumstances.
- Yes, if birth defect and dealth rates increased beyond what has already been observed.
- Yes, if adult death rates increased beyond what has already been observed.
- Yes, under other circumstances - Please describe: ____________________

Please rank your preference for the following mosquito control mechanisms you MOST support for use in the Caribbean in order from ONE (most preferred) to THREE (least preferred). You may only use each number rank once, therefore you must put the three options in order of preference from ONE (most preferred) to THREE (least preferred).

______ Fogging and pesticide spraying in public places

______ Release of genetically modified mosquitoes to reduce populations

______ Personal use of insect repellents/bug sprays and protective clothing

Please rank your preference for the following mosquito control mechanisms you MOST support for use in the United States in order from ONE (most preferred) to THREE (least preferred). You may only use each number rank once, therefore you must put the three options in order of preference from ONE (most preferred) to THREE (least preferred).

______ Fogging and pesticide spraying in public places

______ Release of genetically modified mosquitoes to reduce populations

______ Personal use of insect repellents/bug sprays and protective clothing

At this point in time, how likely are you to avoid travel to the following locations in the coming 12 months due to concerns about the Zika virus on the provided scale of ONE to FIVE?

|  | 1. I would not try to avoid travel to this location. | 2 | 3 | 4 | 5. I am extremely likely to avoid travel to this location. |
| --- | --- | --- | --- | --- | --- |
| The State of Florida |  |  |  |  |  |
| The State of Texas |  |  |  |  |  |
| Puerto Rico |  |  |  |  |  |
| Caribbean Islands |  |  |  |  |  |

Please indicate whether you agree or disagree with the following uses of GMOs.

|  | Strongly Disagree | Disagree | Agree | Strongly Agree |
| --- | --- | --- | --- | --- |
| Grain Production |  |  |  |  |
| Fruit or Vegetable Production |  |  |  |  |
| Livestock Production |  |  |  |  |
| Human Medicine |  |  |  |  |
| Human Health Reasons (i.e. disease vector control) |  |  |  |  |

Which best describes your political affiliation?

- Democratic Party
- Republican Party
- Independent
- None of the above

Which best describes your race?

- White, Caucasian
- Black, African American
- Asian, Pacific Islander
- Mexican, Latino
- American Indian
- Other

Thank you for your time in completing this survey. Your input will strengthen our research and help us obtain more accurate conclusions. If you wish to add any comments please feel free to do so here:
